# Establishment and characterization of the first patient-derived radiation-induced angiosarcoma xenograft model (RT-AS5)

**DOI:** 10.1038/s41598-023-29569-x

**Published:** 2023-02-14

**Authors:** Yvonne M. H. Versleijen-Jonkers, Melissa H. S. Hillebrandt-Roeffen, Marije E. Weidema, Jeroen Mooren, Daniel T. von Rhein, Tessa J. J. de Bitter, Leonie I. Kroeze, Ingrid M. E. Desar, Uta E. Flucke

**Affiliations:** 1 Department of Medical Oncology, Radboud University Medical Center, Geert Grooteplein Zuid 8, 6525 GA Nijmegen, The Netherlands; 2 Central Animal Facility, Radboud University Medical Center, Nijmegen, The Netherlands; 3 Department of Genetics, Radboud University Medical Center, Nijmegen, The Netherlands; 4 Department of Pathology, Radboud University Medical Center, Nijmegen, The Netherlands

**Keywords:** Cancer, Genetics, Oncology

## Abstract

Angiosarcomas are a heterogeneous group of rare endothelial malignancies with a complex, not completely unravelled biology. They encompass primary (sporadically occurring) angiosarcomas of several origins and secondary angiosarcomas, which often arise due to DNA damaging factors including radiotherapy or ultraviolet light exposure. The optimal treatment of metastatic angiosarcomas is unclear and the prognosis is poor. In order to discover novel treatment strategies for angiosarcomas it is important to take the heterogeneity of these tumors into account. For this reason it is also important to have preclinical models available for the different clinical subtypes. Owing to the rarity of angiosarcomas, models are scarce. So far, only five human cell lines of angiosarcomas (all of the scalp after UV exposure) are available worldwide. In this paper we describe a novel established patient-derived xenograft model of a radiotherapy-induced angiosarcoma of the breast. The tumor was characterized by a *MYC* amplification, CD31 and ERG immunohistochemical positivity and was further characterized by using next generation sequencing (TruSight Oncology 500) in combination with the R-package XenofilteR to separate mouse from human sequence reads.

## Introduction

Angiosarcomas (AS) are rare but very aggressive vascular sarcomas with a complex, not completely unraveled biology^1^. They comprise of primary (de novo) and secondary AS, which arise due to DNA damaging factors, like radiotherapy (RT) or ultraviolet (UV) exposure. In case of limited local disease, treatment consists of resection, often combined with radiotherapy. Once progressed to locally advanced or metastatic disease, patients are treated with palliative systemic chemotherapy, mainly paclitaxel or doxorubicin. The only approved targeted drug used for advanced or metastatic disease is the tyrosine kinase inhibitor pazopanib^2,3^.

Despite all therapeutic efforts, the prognosis for AS patients is still very poor, and we recently reported a median overall survival of only 13 months (95% confidence interval of 10–16 months) and a five-year survival rate of 22%^4^. Novel therapeutic approaches are therefore desperately needed for these patients, but progress is hindered because of the diversity and rarity of AS and the lack of models to perform preclinical research.

At the moment there are only five cutaneous (UV-induced) angiosarcoma cell lines available worldwide of which three show tumor growth in mice^5–7^. Because of the large heterogeneity between the different AS subtypes^8^ we aimed to establish a patient-derived xenograft (PDX) model for the radiotherapy-induced subtype, since this is a common AS subtype characterized by its *MYC* amplification^9^.

We therefore implanted four different radiotherapy-induced angiosarcomas of the breast subcutaneously in immunodeficient mice to develop at least one novel patient-derived xenograft model.

## Results

### PDX growth

**Figure 1:**
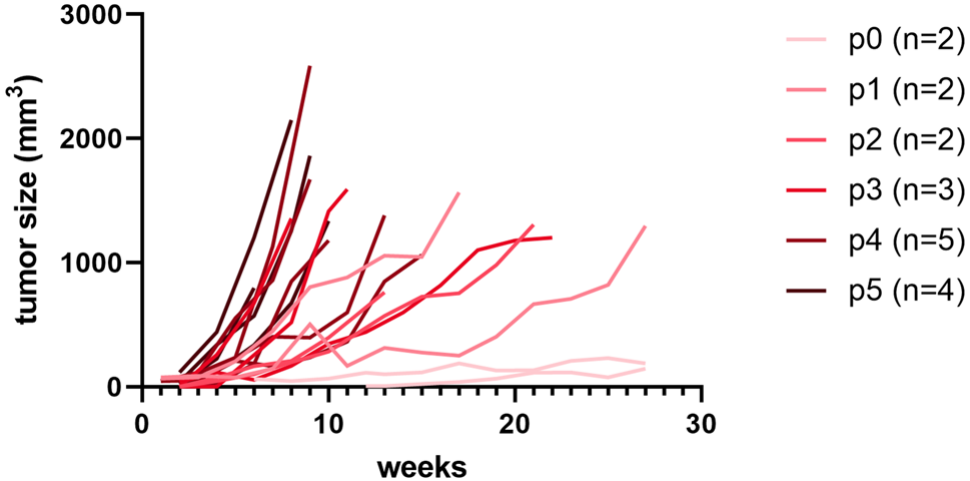
Growth of the model. Growth curves of RT-AS5 tumors in SCID mice in passage 0 to 5. Each line represents a separate tumor. The number of mice per passage varies according to the availability of tumor tissue.

Growth of the PDX was monitored by caliper measurements. Only 1 out of 4 RT-induced AS tumors showed appropriate growth of the tumor in CB-17/lcr-Prkdcscid/Rj SCID mice. This PDX was named RT-AS5. The growth of the tumor accelerated with increasing passages as shown in Fig. 1.

The PDX was derived from a patient who was treated for breast cancer 5 years before the diagnosis of the angiosarcoma which was located in the same breast. The patient was not pretreated with radiotherapy or chemotherapy before resection of the angiosarcoma.

### Characterization of the model

**Figure 2:**
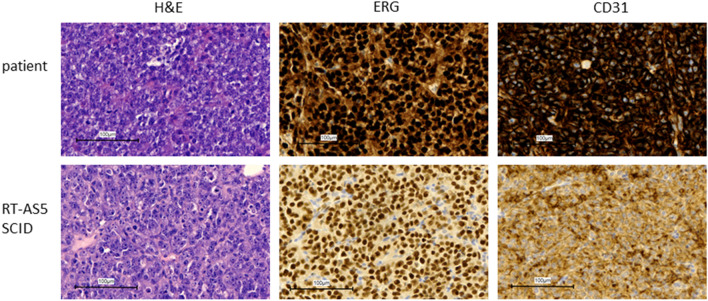
Characterization of the model. H&E, ERG and CD31 staining on patient and PDX material.

**Figure 3:**
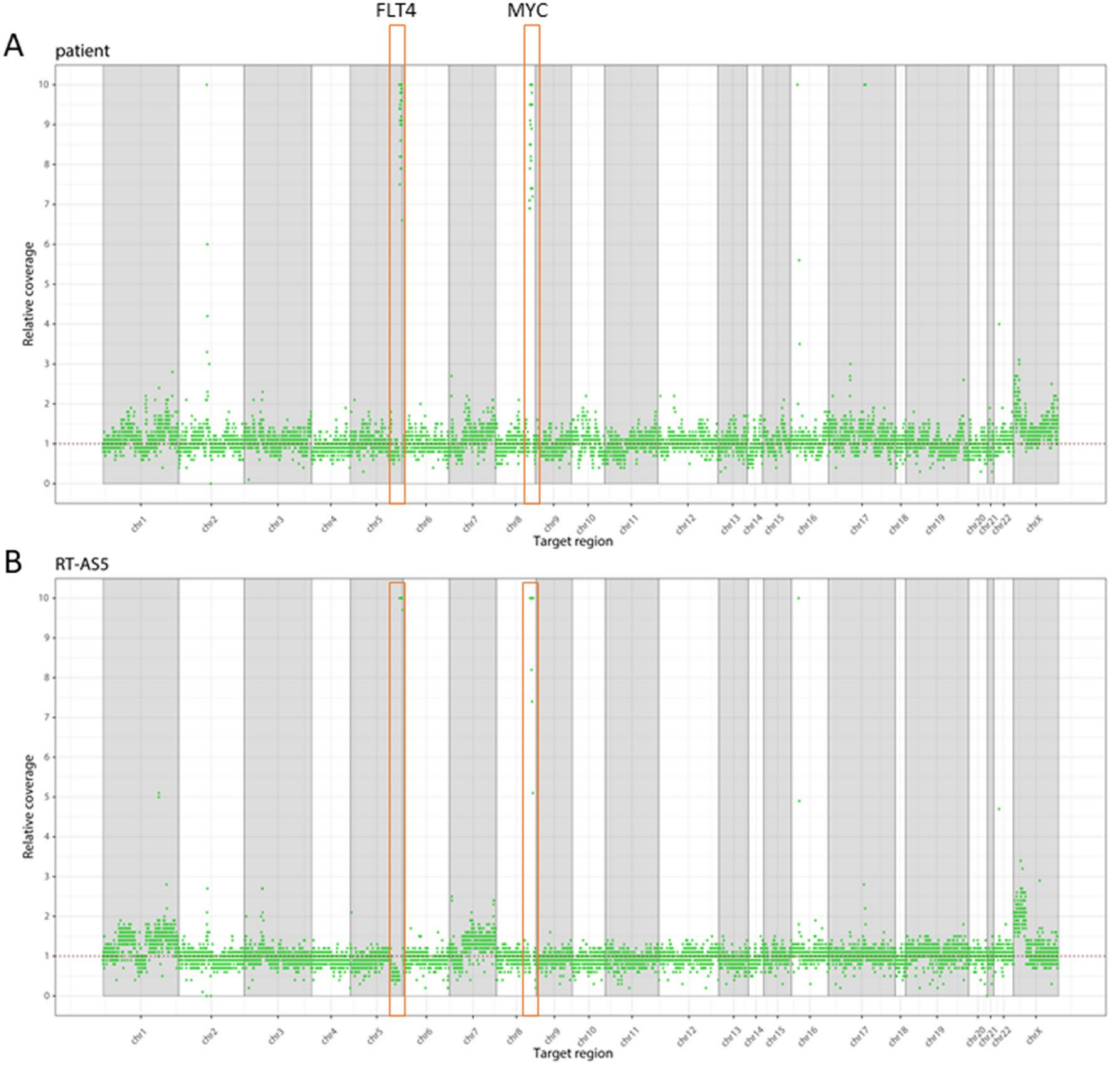
Copy number variations. Copy number variations in patient (**A**) and PDX (**B**) material, including amplifications of MYC and FLT4. Vertical bars represent chromosome 1–22, X and Y. A relative coverage of one corresponds to an equal number of copies as in the set of normal controls (2 copies of a gene region). A relative coverage of > 1 or < 1 indicates additional copies or loss of a copy, respectively.

Immunohistochemical expression of the AS-specific diagnostic markers ERG and CD31 confirmed the AS diagnosis in patient material as well as in dissected RT-AS5 PDX material (Fig. 2; patient and RT-AS5 SCID). Copy number analysis revealed *MYC* and *FLT4* amplification in the RT-AS5 PDX model, as was also shown in the corresponding patient sample (Fig. 3). A large number of genomic variants was detected in the RT-AS5 PDX sample, but after manual filtering most variants appeared to be known polymorphisms. Only 1 variant appeared to be likely pathogenic, concerning a small deletion in *FANCI* (GRCh37: Chr15:g.89805050_89805053del). The original patient sample was subsequently analyzed by TSO500 panel sequencing to validate our findings and this analysis revealed identical non-pathogenic variants (shown in Table 1) and the same deletion in *FANCI* being the only likely pathogenic variant identified in this sample.

**Table 1:** Non-pathogenic variants found in both patient and PDX material.

Gene	GRCh37 sequence
*TET2*	4:g.106196770G > A
*ATM*	11:g.108138003 T > C
*ANKRD26*	10:g.27324401 T > G
*FGF1*	5:g.141993631C > T
*NCOR1*	17:g.15973774 T > G
*FLT1*	13:g.28971113 T > C
*GATA2*	3:g.128204960G > C
*GEN1*	2:g.17954397G > A
*NOTCH1*	9:g.139400320G > A

### Cryopreserved material

**Figure 4:**
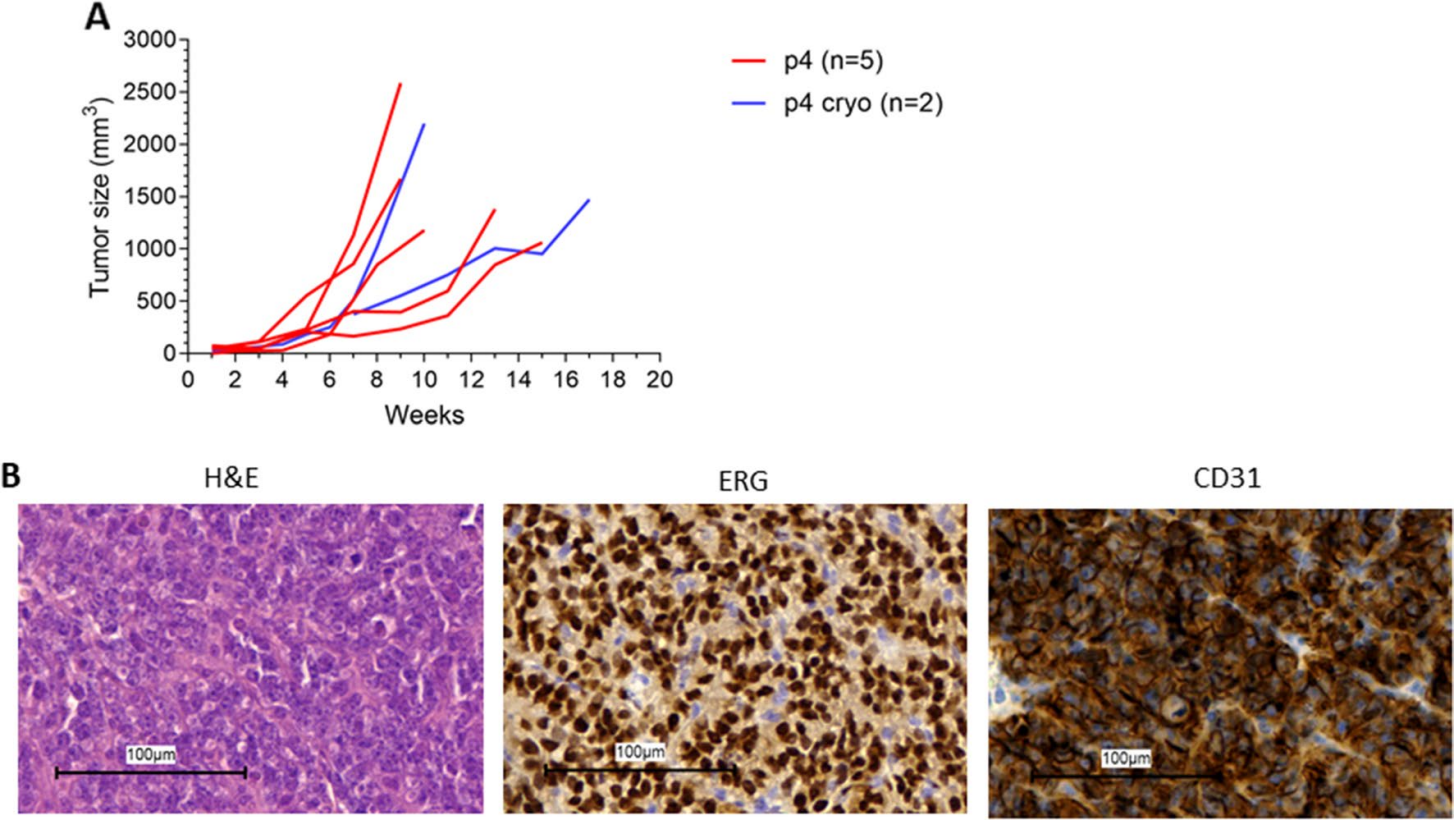
Growth of cryopreserved PDX material. (**A**) Growth curves of PDX in mice in the original passage 4 (red lines) and mice implanted with cryopreserved PDX material of the same passage (blue lines). Each line represents a separate tumor. (**B**) H&E, ERG and CD31 staining on cryopreserved PDX material.

At every passage we collected material in medium/DMSO at − 80 °C. Tumor material from passage 4 was implanted in 2 mice to test the growth rate from this frozen material. Growth curves are compared with the fresh passage 4 as depicted in Fig. 4. Growth rate was almost equal to the fresh passage of material, and AS diagnosis was confirmed by the expression of the diagnostic biomarkers ERG and CD31, which shows the usability of frozen material and thus the possibility to exchange PDX material with other research labs.

### Growth in BALBc nude mice

**Figure 5:**
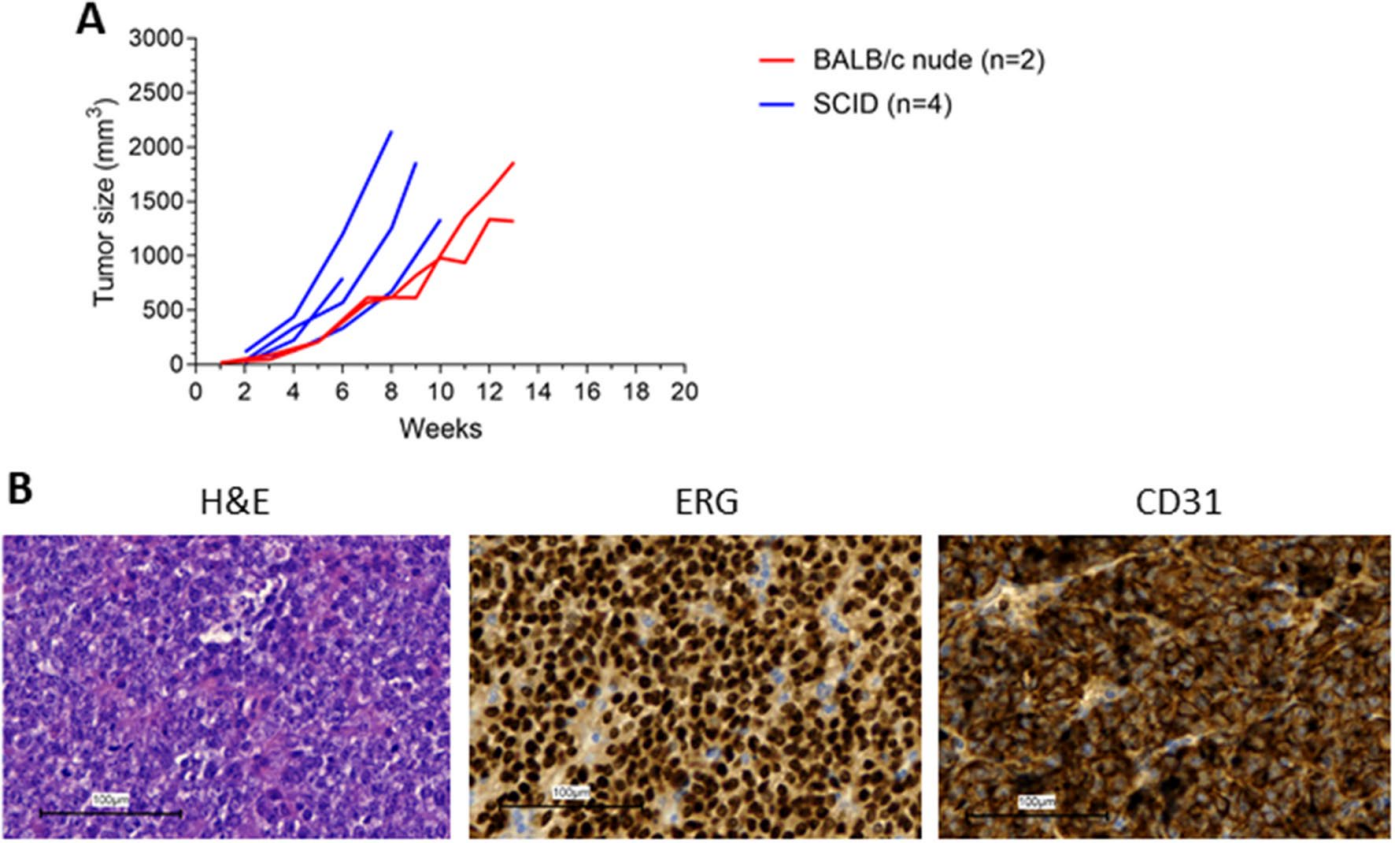
Growth in BALB/c nude mice. (**A**) Growth curves of PDX passage 5 in SCID mice versus BALB/c nude mice. Each line represents a separate tumor. (**B**) H&E, ERG and CD31 staining on PDX material implanted in BALB/c nude mice.

Tumors were at first grown in CB-17/lcr-Prkdcscid/Rj SCID mice because they are expected to grow well in these severe immunodeficient mice, with the absence of functional T and B cells. However, these mice are more sensitive to infections compared to for instance BALB/cAnNRj-*Foxn1*^*nu*^*/Foxn1*^*nu*^ mice which lack a thymus and are thus unable to produce T cells. We therefore also tried to grow the RT-AS5 tumor in the less sensitive BALB/c nude mice. Figure 5 shows that the tumors also show growth in these mice. The AS diagnostic markers confirm the diagnosis.

## Discussion

So far, only five human angiosarcoma cell lines are available worldwide. They are derived from sun-damaged skin with three having shown growth in mice^5–7^. Additional human PDX models have not been described so far. Because of the different AS genesis^8^, we need additional models of the various subgroups, including the rather common radiotherapy-induced AS, to perform further translational research.

Although cell line-derived models are commonly easy to create, there are numerous limitations since these models do not represent the distinctive features of a patient. PDX models are established through the engraftment of tissue from a patient’s tumor into an immunodeficient mouse, which preserves cell–cell interactions and the tumor microenvironment^10^.

We have tested the growth of our RT-AS5 model after cryopreservation to make sure that the tumor will be able to grow after storage and transport. This makes it possible to share the model with interested researchers in collaboration.

Our model is suitable for testing the efficacy of various targeted treatments based on the genomic aberrations that we detected including *MYC* and *FLT4* amplification which have been described previously in AS, especially in the RT-induced subgroup^11^. The MYC oncoprotein itself cannot be targeted by clinically available drugs, however its transcription can be modulated by BET inhibitors^12,13^. The *FLT4* gene encodes for the vascular endothelial growth factor receptor 3 (VEGFR3)^14^. Guo et al. reported three cases of *FLT4* amplified secondary AS, which showed complete or partial response to sorafenib, a multikinase inhibitor^15^. *FLT4* amplified tumors might also benefit from other VEGFR inhibitors and this model could be used for further evaluation of the effects of these inhibitors. Thibodeau et al. described a mutational pattern in radiation-induced breast angiosarcomas which was associated with genes involved in DNA repair^16^. They performed next generation sequencing on 13 RT-induced AS samples using a targeted panel of 160 cancer related genes and observed that all 13 cases had at least 2 genes affected in DNA damage pathways. In total 17 different genes in the DNA damage response (DDR) system contained a variant in one of the investigated cases, i.e*. ARID1A, ARID2, ATM, BRCA1, BRCA2, BRIP1, CHEK1, CHEK2, FANCA, FANCD2, FANCE, MSH2, MSH6, PBRM1, RB1, SMARCA4, SMARCB1* and *TP53*. They conclude that the DDR pathway may prove to be a promising target in these tumors. In our PDX model we also found a variant in a gene playing a role in the DDR system, i.e. *FANCI*, making this model also relevant for testing the effects of DDR inhibition. Further pharmacogenomic studies in angiosarcoma and other soft tissue sarcomas are important to determine the presence of this variant.

## Conclusion

This is the first described and fully characterized radiotherapy-induced angiosarcoma model which is suitable for further exploration of potential targeted treatments for AS.

## Methods

### Ethical approval for the use of human tissue

Anonymized tumor tissues from angiosarcoma patients undergoing surgery were collected with informed consent. Ethical approval was obtained from the local certified Medical Ethics Committee of the Radboudumc, Nijmegen, The Netherlands (File number 2016-2686). All experiments were performed in accordance with relevant guidelines and regulations.

### Ethical approval for the use of animals

All applicable (inter)national and institutional guidelines and regulations for the care and use of animals were followed. All procedures performed were in accordance with the ethical standards of the animal ethical committee of the Radboud University, Nijmegen, The Netherlands (Project# 2015-0109). The study is reported in accordance with the Animal Research Reporting of In Vivo (ARRIVE) guidelines.

### In vivo growth

Female CB-17/lcr-Prkdcscid/Rj SCID or BALB/cAnNRj-*Foxn1*^*nu*^*/Foxn1*^*nu*^ mice from Janvier labs (6–8 weeks old) were subcutaneously implanted with small tumor pieces (~ 3 mm in diameter) under anaesthesia via inhalation of isoflurane (2.5–3.0%). Tumor growth was monitored by biweekly caliper measurements in three dimensions (length (l), width (w) and height (h); all maximum diameter). Tumor size was calculated using the formula: 4/3π x l/2 × w/2 × h/2. Mice were euthanized by cervical dislocation at a maximal tumor volume of 2 cm^3^. Tumors were resected and used for passage into additional mice or for model characterization.

From previous experiments we knew that some xenografts take a long period of time to start growing. However, if after 6 months no tumor growth was detected the model was considered unsuitable.

### Use of cryopreserved material

At each passage tumor pieces were stored in Medium 199 (ThermoFisher) with 10% DMSO at − 80 °C. We implanted these pieces again in two mice to check if we were able to regrow the tumor pieces in order to be able to; (1) stop passaging the tumors when no experiments were planned in order to spare mice, and (2) share the material with other researchers. To thaw tumor pieces, aliquots were warmed to 65 °C in a heated water bath and tumor tissue was washed in complete Medium 199 (without DMSO) and immediately implanted in mice.

### Characterization of the model

Hematoxylin and Eosin (H&E) staining and immunohistochemical stainings for the angiosarcoma specific diagnostic markers ERG and CD31 were performed to characterize the model^17^. Immunohistochemical stainings were performed in the Lab Vision Autostainer 360 (ThermoFisher Scientific) by using the EnVision FLEX, pH High Link Kit (Dako) and monoclonal rabbit anti-ERG (1:500, clone EPR3864, Abcam) or monoclonal mouse anti-CD31 (1:100, clone JC70A, Dako).

TSO500 panel-based sequencing was performed as described previously to analyze genomic aberrations that were present in the model^18,19^.

In brief, genomic DNA was isolated from formalin-fixed paraffin-embedded (FFPE) tissue. Library preparation was performed using the hybrid capture-based TSO500 library preparation kit (Illumina) following the manufacturer’s protocol.

Libraries were sequenced on a NextSeq 500 (Illumina). Sequence data were processed and analyzed by the TruSight Oncology 500 Local App version (Illumina). Unique molecular identifiers (UMIs) were used in the analysis to determine the unique coverage at each position. Coverage tables and a variant call file for single- and multiple-nucleotide variants, including number and percentage of variant alleles, were provided. The R-package Xenofilter was used to exclude mouse sequences that disturbed the analysis of the patient-derived xenograft tissue^20^.

Genomic variants were filtered by excluding: (1) variants not overlapping with exons and splice site regions (− 8/ + 8) except those in the TERT promoter region, (2) synonymous variants, unless located in a splice site region, (3) variants present with a frequency > 0.1% in the control population represented in The Exome Aggregation Consortium (ExAC) version 0.2, and (4) variants with a variant allele frequency of < 5%.

Identified variants were interpreted using the software Alamut visual version 2.13 and in addition, the aggregated knowledge-based tools, ClinVar, OncoKB and InterVar were used to review specific variants. Variants were manually analyzed and classified based on the predicted pathogenicity into 5 classes: class 1, not pathogenic; class 2, unlikely pathogenic; class 3, variant of unknown significance; class 4, likely pathogenic; and class 5, pathogenic. The interpretation of pathogenicity for variants in tumor suppressor genes (TSGs) was based on three prediction tools (sorting intolerant from tolerant (SIFT), Polyphen-2 and Align-Grantham Variation Grantham Deviation (Align-GVGD).

Amplifications were called based on median coverage normalization as previously described^18^. A relative coverage ≥ 3 was considered gene amplification. The number of gene copies was estimated by using the relative coverage corrected for the percentage of tumor cells in the sample.

The corresponding original patient sample was subsequently analyzed by TSO500 panel-based sequencing to validate the results of the PDX model.

## Data Availability

Binary Alignment Map (BAM) and corresponding (annotated) variant call format (VCF) files of the patient cannot be publicly shared under the obtained institutional review board approval, since the patient was not consented to share raw sequencing data beyond the research and clinical terms. However, all variants assessed as (potentially) clinically relevant are presented in this paper. The data generated and analyzed during the current study are available from the corresponding author on reasonable request and upon a data usage agreement.
