# Author Correction: Establishment and characterization of the first patient-derived radiation-induced angiosarcoma xenograft model (RT-AS5)

**DOI:** 10.1038/s41598-025-07810-z

**Published:** 2025-07-10

**Authors:** Yvonne M. H. Versleijen‑Jonkers, Melissa H. S. Hillebrandt‑Roeffen, Marije E. Weidema, Jeroen Mooren, Daniel T. von Rhein, Tessa J. J. de Bitter, Leonie I. Kroeze, Ingrid M. E. Desar, Uta E. Flucke

**Affiliations:** 1 Department of Medical Oncology, Radboud University Medical Center, Geert Grooteplein Zuid 8, 6525 GA Nijmegen, The Netherlands; 2 Central Animal Facility, Radboud University Medical Center, Nijmegen, The Netherlands; 3 Department of Genetics, Radboud University Medical Center, Nijmegen, The Netherlands; 4 Department of Pathology, Radboud University Medical Center, Nijmegen, The Netherlands

Correction to: *Scientific Reports* 10.1038/s41598-023-29569-x, Published online 14 February 2023

The original version of the Article contained an error in Fig. 2. As a result of an error during figure assembly, the H&E staining for the patient panel in Fig. 2 was a duplication of the H&E staining panel in Fig. 5.

The original Fig. 2 and accompanying legend appear below.

**Fig. 2:**
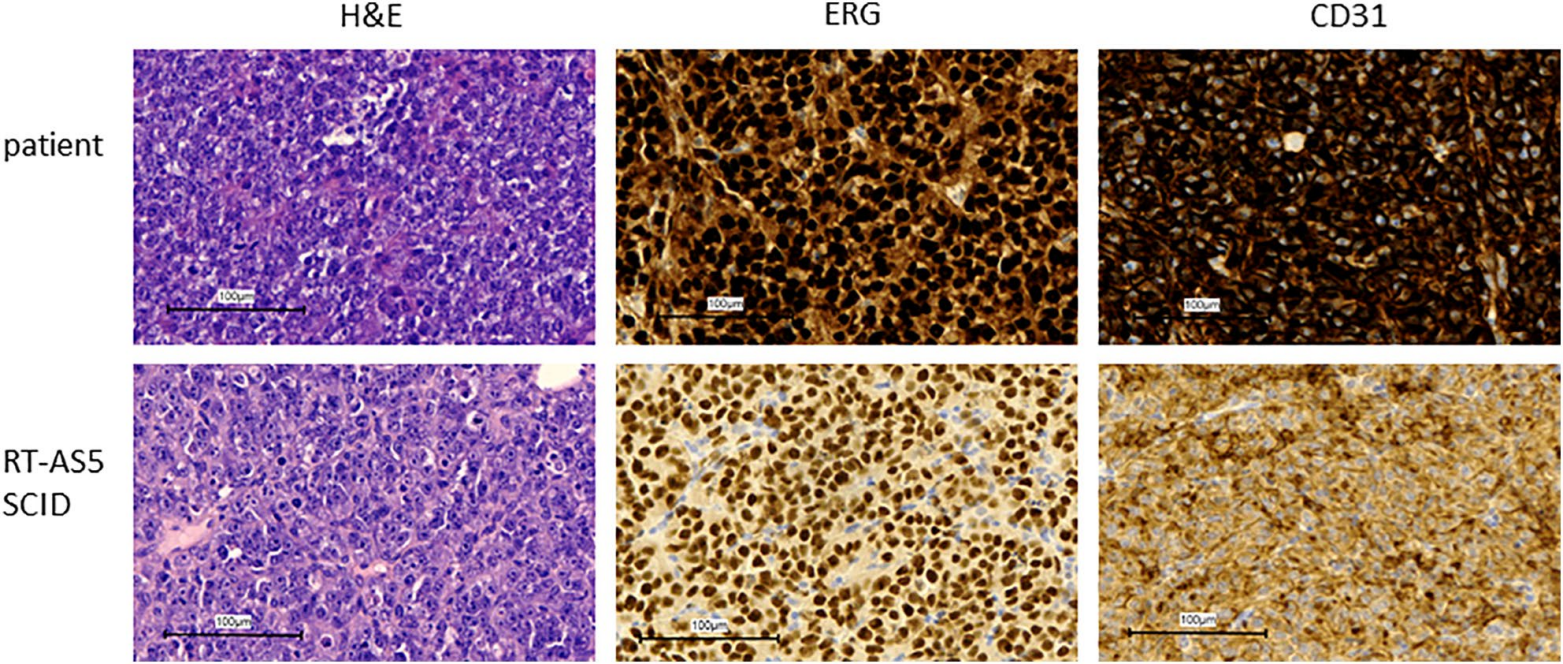
Characterization of the model. H&E, ERG and CD31 staining on patient and PDX material.

The original Article has been corrected.

